# The development and validation of scales to measure the presence of a teachable moment following a cardiovascular disease event

**DOI:** 10.1016/j.pmedr.2022.101876

**Published:** 2022-06-27

**Authors:** Michelle Brust, Winifred A. Gebhardt, Nadine A.E. van der Voorde, Mattijs E. Numans, Jessica C. Kiefte-de Jong

**Affiliations:** a Department of Public Health and Primary Care/Health Campus The Hague, Leiden University Medical Center, The Hague, The Netherlands; b Department of Health, Medical and Neuropsychology, Leiden University, Leiden, The Netherlands

**Keywords:** Teachable moments, Lifestyle, Prevention, Questionnaire development, Validation, Health communication

## Abstract

Better conceptually-driven research is necessary to learn more about 1) the characteristics of life events as teachable moments (TMs) and 2) the potential of life events to evoke lifestyle change intention (LCI). This study aimed to develop and validate two scales for the purposes of TM research in the context of cardiovascular disease (CVD): the *CardiacTM* and *CardiacLCI-*scales. After the initial development of items based on a theoretical framework and literature search, six experts rated the content validity of both scales as sufficient. The item list was further adjusted after think-aloud sessions with two CVD patients. The resulting scales were presented online in a cross-sectional survey, which yielded 625 responses of Dutch CVD patients (June 2020). To test construct validity, we conducted Exploratory Factor Analysis (EFA) with Varimax rotation on a random split-half of the sample (n = 300) and evaluated the factor structure with Confirmatory Factor Analysis (CFA) on the holdout sample (n = 325). EFA and CFA on the CardiacTM-scale (α = 0.88) revealed a 28-item six-factor structure explaining 61.0% of the variance, with adequate goodness-of-fit statistics (CFI = 0.87; TLI = 0.85; SRMR = 0.07) and internally reliable factors (Affective impact, Risk CVD, Changed self-concept, CVD group identity, Risk non-communicable disease, Anticipated regret). The CardiacLCI-scale (α = 0.81) revealed an 11-item two-factor structure explaining 51.5% of the variance, with adequate model fit (CFI = 0.92; TLI = 0.90; SRMR = 0.08) and internally reliable factors (Event-related lifestyle change and General healthy lifestyle). The scales may be used to expand knowledge around life events as TMs and to support conversation regarding lifestyle after cardiac and other life events.

## Highlights

• Investigating cardiac events as teachable moments is a promising venture.
• Validated measures to conduct research on teachable moments are lacking.
• We developed scales that demonstrate validity and reliability.
• The scales could be employed in research on teachable moments and behavior change.
• Clinicians could use the scales to foster conversation around lifestyle.

## 1 Introduction

Cardiovascular diseases (CVD) are the leading cause of mortality both in the Netherlands and worldwide (Roth et al., 2020). The course of cardiometabolic health is heavily influenced by the ability of an individual to change unhealthy behaviors such as smoking, unhealthy dietary uptake, and insufficient physical exercise (IHME) IfHMaE. Findings from the Global Burden of Disease Study, 2017). Nonetheless, the prevalence of unhealthy behaviors is increasing, and even amongst those wishing to live a healthier life, the adoption and maintenance of risk-reducing health behaviors is often a major challenge ([3]). Within the field of health behavior change, research has identified life situations that may help instigate risk-reducing changes in behavior. These situations, referred to as ‘teachable moments’ (TMs), often occur in the time window following an important life or health event, and individuals may be more willing to optimize their lifestyle following a TM (McBride et al., 2003, Lawson and Flocke, 2009, Flocke et al., 2014, Brust et al., 2021). Because CVD events have been linked to lifestyle changes such as quitting smoking (Tofler et al., 2015), scholars suggest that a potential TM following a cardiac event may induce the adoption of healthier lifestyle behaviors among affected patients (Tofler et al., 2015, Coull and Pugh, 2021).

Based on existing reports, investigating cardiac events as potential TMs appears promising. It is logical to suppose that individuals might be more receptive to health behavioral messages during certain life events, a supposition confirmed in a study of cancer screening (Stevens et al., 2019). Health-promoting interventions during or close to TMs might therefore be better received and thus more effective in achieving improved health behavior outcomes (Flocke et al., 2014, Cohen et al., 2011, Flocke et al., 2014, Karvinen et al., 2015). Potential TMs around cardiac events consequently represent an important and promising opportunity to convey lifestyle advice at the optimal moment in a healthcare trajectory. The TM concept is, however, still insufficiently developed (McBride et al., 2003, Lawson and Flocke, 2009). A better appreciation of the factors mediating TMs could increase our understanding of the nature of motivation to change (Schnoll et al., 2013), as well as help guide health communication tailored to related underlying cognitions.

The experienced significance of a life event for an individual is likely to be determined by the way an event is cognitively interpreted (McBride et al., 2003, Gropper et al., 2020). This interpretation in turn determines its impact on intentions towards behavioral change and actual changes in behavior (McBride et al., 2003, Kark Smollan, 2006, Schlossberg, 2011). According to a conceptual framework proposed by McBride et al. (McBride et al., 2003), an effective TM is characterized by three key cognitive characteristics: 1) an increased perception of a person’s personal risk, 2) an affective or emotional impact of an event, and 3) a redefinition of a person’s self-concept. This framework is employed as the basis of a substantial part of TM research, and efforts have been made to empirically test the applicability of the framework (McBride et al., 2017, McBride et al., 2008). There is, however, major inconsistency in the way scholars measure increased risk perception, affective impact, and changed self-concept (Lawson and Flocke, 2009). An example of this inconsistency can be found in two studies by McBride et al. (McBride et al., 2017, McBride et al., 2008) in which being diagnosis of cancer was explored as a TM. The former study assessed affective impact by asking patients to rate their concern regarding colon cancer development (McBride et al., 2008), while the latter study assessed the Positive and Negative Affect Scale (PANAS) (Thompson, 2007) as a measure of affective impact (McBride et al., 2017). Naturally, inconsistency in methodologies impacts the comparability of studies that explore potential TM events.

Differences in research methodology within TM research highlight a need for better designed and conceptually-driven studies when exploring life events as potential TMs (McBride et al., 2003, Lawson and Flocke, 2009, Rabin, 2009). However, validated measures specifically designed for this purpose are still lacking. The main aim of the present study was therefore to develop and validate two scales: 1) the *Cardiac Teachable Moment Framework* scale (CardiacTM), which aims to assess whether a cardiac event fullfills the TM criteria of risk perception, affective impact, and changed self-concept, and 2) the *Cardiac-induced Lifestyle Change Intention* scale (CardiacLCI), which aims to assess whether a cardiac event actually induced a subsequent lifestyle change intention (LCI) in affected patients. This study applied the principles of scale development and validity testing as recommended by Boateng et al. (Boateng et al., 2018), and consisted of: 1) item development, 2) construct development, and 3) construct evaluation.

## 2 Methods

### 2.1 Step 1: Item development

#### 2.1.1 Identification of domains and item generation

Applying the guidelines formulated by Boateng et al. (Boateng et al., 2018), the development of our questionnaires began with a thorough exploration of the constructs of interest. Hence, we first searched literature to explore subdomains that comprised our constructs CardiacTM (including risk perception, affective impact and changed self-concept(4)) and CardiacLCI. The search was performed in PubMed and Web of Science, using keywords described in Supplemental Material 1. Once the subdomains were defined, we generated items pertaining to these subdomains (Boateng et al., 2018, Hinkin, 1995). Whenever possible, items of existing validated measures were used as the basis for our new scales. For those constructs that lacked comparable measures, items were generated during brainstorm sessions involving the researchers (NAEV, MEK, MB). To maintain consistency, all items had to be unipolar and formulated as statements that could be assessed using a 7-point Likert scale (ranging from strongly disagree to strongly agree, as these anchors demonstrate the most equal conceptual distance (Casper et al., 2020).

#### 2.1.2 Modification of items by an expert panel and a target group

The second step in item development was to administer the scales to an expert panel in order to assess content validity (Boateng et al., 2018). Six experts working in cardiac care were asked to provide qualitative feedback on the items and to rate the clarity and relevance of the items using the Content Validity Index (I-CVI) (from 1 = an irrelevant item to 4 = an extremely relevant item) (Zamanzadeh et al., 2015). A I-CVI of ≥ 0.80 is recommended for sufficient content validity of an item (Zamanzadeh et al., 2015).

After assessing content validity, the final step in item development was to pre-test the adjusted scales using the think-aloud method (Charters, 2003). In this method, members of the target population were instructed to think out loud when filling in the scales. Two patients who experienced a stroke 6 months or 2 years prior to the think-aloud session, respectively, participated the think-aloud session in the (online) presence of a researcher (NAEV). The thoughts and comments of the patients on the items was subsequently used to remove or adapt items that were unclear or difficult (Charters, 2003).

### 2.2 Step 2 and 3: Construct development and construct evaluation

#### 2.2.1 Procedure, participants and measures

After the initial items were developed and revised, the next step was to determine psychometric properties of the scales by asking a larger number of CVD patients to fill out the questionnaire. The questionnaire in this cross-sectional survey study was distributed online (www.Qualtrics.com) and comprised the items of our newly developed scales plus additional measures to assess convergent validity (Bolarinwa, 2015). To explore convergent validity of the ‘risk perception’ items, we included the Perceived risk of a heart attack/stroke subscale from the ABCD questionnaire (Woringer et al., 2017), which consists of 8 items (e.g. I feel I will suffer from a heart attack or stroke sometime during my life) with response options ranging from 1 = strongly agree to 4 = strongly disagree. Regarding the ‘affective impact’ items, we first included the 3-item ultra-brief form of the Penn State Worry Questionnaire (PSWQ) (Berle et al., 2011) (e.g. Once I start worrying, I cannot stop), with response options ranging from 1 = not at all typical to 5 = very typical. This shorter PSWQ version showed comparable internal consistency to the normal PSWQ, which is regarded as the gold standard when assessing worry (Berle et al., 2011). We also included the Negative Affect subscale from the PANAS short-form, a frequently used scale for assessing emotions, in which participants could indicate the extent to whether they experienced five negative emotions (e.g. Upset) (Thompson, 2007). For the ‘self-concept’ items, we included the Acceptance subscale of the Illness Identity Questionnaire (IIQ) (Oris et al., 2016) due to its relatedness to the construct. We did not explore convergent validity of the CardiacLCI-scale, because measures to capture event-induced LCI are currently lacking. All measures were initially translated to Dutch using a back-translation process with three bilingual researchers, in which original English items were first translated to Dutch by the first researcher, then back-translated to English by the second researcher, after which a third researcher compared meaningful differences between the original and back-translated items and decided on approval of the translation (Brislin, 1970).

The anonymous link to the questionnaire was distributed through the Dutch Heart Foundation website and via an e-mail invitation to members of Harteraad, the largest Dutch CVD patient organization. Individuals were eligible to participate if they were 18 years or older, were able to read Dutch, and had suffered from any form of heart problem(s) for which they had been hospitalized. After providing online informed consent, participants were requested to keep their most recent cardiac event in mind when filling out the remaining questionnaire. The study was approved by the Medical Ethics Committee of Leiden University Medical Center in April 2020 (METC-nr 18–112).

#### 2.2.2 Statistical analysis

For both scales, analyses were conducted in several steps, comprising factor validity, convergent validity (only CardiacTM-scale) and reliability (Bolarinwa, 2015). We initially explored the normality of all items and removed items that visually demonstrated outliers in QQ-plots or had skewness values of > 2 or kurtosis values of > 7 (Kim, 2013). To deal with overfitting when using the same dataset for both Exploratory Factor Analysis (EFA) and Confirmatory Factor Analysis (CFA), we randomly divided our dataset in two mutually independent halves (Boateng et al., 2018). Using the first half of the dataset, we assessed the suitability of the data for EFA based on the Kaiser-Meyer-Olkin Measure of Sampling adequacy (KMO) and Bartlett’s test of sphericity (Watkins, 2018, Kaiser, 1974). We then conducted EFA and extracted factors using an orthogonal (Varimax) rotation approach (Tabachnick and Fidell, 2007). We inspected the scree plot to decide on the number of factors to be extracted and carried out iterations in EFA to identify core constituent items in each factor. Cross-loading items, items with loading ≤ 0.30, and/or items with loadings with a<0.20 difference between factors were deleted at each iteration (Howard, 2016). Using the second half of the dataset, we evaluated the factor solution with CFA with maximum likelihood estimation. Standardized factor loadings were deemed as unacceptable below 0.10 (Nagy et al., 2017). The Chi-square (χ^2^)-index is often used to evaluate model fit, but is highly sensitive to large sample size (Lt and Bentler, 1999). Therefore, we additionally explored two incremental fit indices (the Comparative Fit Index (CFI) and Tucker-Lewis Index (TLI)) as well as one residuals-based fit index (the Standardized Root Mean Square Residual (SRMR)) (Lt and Bentler, 1999). The CFI and TLI should exceed 0.90 (Kline, 2015) and the SRMR should be lower than 0.08 to indicate good model fit (Lt and Bentler, 1999). The resulting factors were additionally tested for convergent validity with the Pearson product-moment correlation, with < 0.30 demonstrating low, 0.30–0.50 medium, and > 0.50 high convergent validity (Cohen, 1988). Lastly, the internal reliability of all factors was assessed using Cronbach’s α coefficients with α ≥ 0.70 as the threshold indicating sufficient reliability and α ≥ 0.80 demonstrating good reliability (Cortina, 1993).

## 3 Results

### 3.1 Step 1: Item development

#### 3.1.1 Identification of domains and item generation

The subdomains identified are presented in Table 1 and the complete results of the literature search are elaborated in Supplementary Material 1. We selected and drafted 74 initial items to fully measure the broad construct CardiacTM and 16 items to measure the construct CardiacLCI. All items, including references to the source questionnaires, are provided in Supplementary Material 2.

**Table 1:** Identification of subdomains.

**CardiacTM-scale**	**CardiacLCI-scale**
Risk perception	Lifestyle change intention
Perceived susceptibility and severity of CVD	Impact of event on lifestyle
Perceived susceptibility and severity of NCD	
Perceived relative risk	
Increase in risk perception after cardiac event	
Affective impact	
Level of worry	
Negative affect	
Self-concept	
Social role	
Perceived stigmatization	
Identity and lifestyle	
Future/possible self	
Feeling of self-worth	
Body image	

*Note:* CVD = cardiovascular diseases; NCD = non-communicable diseases.

### 3.2 Modification of items by expert panel and target group

#### 3.2.1 Cardiac teachable moment framework scale

The I-CVI for the clarity and relevance of items ranged from 0.50 to 1 (Supplementary Material 3). Items 4, 14, 15, 40–42, 55–58, 68, and 73 were deleted based on I-CVI values < 0.80. Qualitative feedback from experts resulted in the revision of items 6, 8, 16, 17, 19–21, 45, 48, 64, 69 and 71 to improve readability, and the addition of two items to better capture the constructs (e.g. Since my cardiac event, I feel more often down).

The qualitative feedback from the patients during the think-aloud sessions resulted in the additional deletion of item 3, 22, 23, 42 and 67 because they were difficult to understand or overlapped with similar items. Furthermore, it resulted in changes to the phrasing of items 9, 13, 18, 25, 28 and 67 in order to increase readability, such as the removal of double negatives. For example, item ‘I think my chances that I will experience lifestyle-related diseases in the next ten years are low’ was rephrased into ‘I think my chances (…) are high’. Additionally, all items that contained the phrase ‘since my heart incident’ were rephrased into ‘due to my heart incident’, in order to optimally capture whether a change could be annotated to the cardiac event itself. The resulting 59-item scale is presented in Supplemental Material 4.

#### 3.2.2 Cardiac-induced lifestyle change intention scale

The I-CVI rating for clarity and relevance caused the elimination of three items (Lawson and Flocke, 2009, Cohen et al., 2011, Gropper et al., 2020) (Zamanzadeh et al., 2015) from the CardiacLCI-scale. Qualitative feedback by experts led to the modification of items 10 and 13 in order to further improve the scale. Following the expert’s recommendations, we added two items (e.g. I sometimes think about improving my lifestyle).

The think-aloud sessions on the improved list of 15 items led to a final revision in which the formulation of items 1, 2 and 8 was adjusted to increase readability. Items 9 and 14 were removed to lower possible annoyance due item repetition. Finally, two items were added because one patient felt there was no opportunity to report the conditions *already living a healthy lifestyle* and be*ing tempted by unhealthy behaviors*. The resulting 15-item scale is presented in Supplemental Material 4 .

### 3.3 Step 2 and 3: Construct development and construct evaluation

#### 3.3.1 Patient characteristics

The link to the online questionnaire was send to 2606 patients who experienced a cardiac event, of which 625 patients provided us with valid responses (24%). Sociodemographic characteristics of the sample are presented in Table 2. The average age of our sample was 58.5 (*SD* = 9.9), and the majority of our sample was male (63%), lived together with a partner (73%), had completed higher education (46%), and was hospitalized for heart rhythm disorder as their most recent cardiac event (29%). The dataset was initially divided random halves to perform EFA (n = 300) and CFA (n = 325).

**Table 2:** Sociodemographic characteristics of the complete sample (n = 625).

**Characteristic**	**Mean (SD)**	
**Age**	58.5 (9.9)	
	**Frequency (n)**	**Percentage (%)**
**Gender**		
Female/male	228/394	37/63
**Living situation**		
Living alone/cohabiting	213/604	25/73
**Education**		
Low/middle/high	127/208/290	20/33/46
**Most recent cardiac event**		
Angina pectoris	112	18
Myocardial infarction	126	20
Heart failure	78	13
Heart valve disease	44	7
Heart rhythm disorder	179	29
Cardiomyopathy	21	3
Vascular disease	109	17
Stroke	19	3
**Time from most recent event**		
<6 months ago	119	19
6–12 months ago	75	12
1–3 years ago	152	25
3–10 years ago	198	32
>10 years ago	77	12

*Note.* Total percentages can deviate from 100% due to rounded numbers. Low education = no, elementary or vocational education; middle education = higher general or secondary vocational education; high education = higher professional and scientific education.

#### 3.3.2 Cardiac teachable moment framework scale

##### 3.3.2.1 Exploratory factor analysis

Of the 59 items, presented in Supplementary Material 4, two ([3]) were removed based on skewness/kurtosis values < 2. The KMO (0.84) and significant Bartlett’s test of sphericity (χ2(1378, N = 300) = 6617;p < 0.001) indicated that data from the first half of the dataset were acceptable for factor analysis (Watkins, 2018). Next, seventeen items (McBride et al., 2003, Lawson and Flocke, 2009, Brust et al., 2021, Karvinen et al., 2015, Kim, 2013, Watkins, 2018, Bandura, 1998, Stets, 2020, Becker, 1974, Olson, 2010, Nagy et al., 2017, Lt and Bentler, 1999, Kline, 2015, Cohen, 1988, Fotuhi et al., 2013, Chiou and Wan, 2007, Oliveira et al., 2019) were removed from the dataset because they consistently showed communities below < 0.30, indicating that they possibly not load sufficiently on any factor. The new EFA showed that nine factors had eigenvalues over Kaiser’s criterion 1. Based on the scree plot (Supplementary Material 5), a three to seven factor solution seemed suitable. Further inspection of the item loadings revealed that six factors best fitted the data. Finally, twelve items (Kark Smollan, 2006, Schlossberg, 2011, McBride et al., 2017, Rabin, 2009, Hinkin, 1995, Kaiser, 1974, Festinger, 1957, Orcullo and Teo, 2016, Cortina, 1993, Peters et al., 2019, Alqahtani et al., 2020, Bergner and Holmes, 2000) were iteratively removed iteratively due to cross-loading or loading on the wrong factor. The resulting six-factor structure (Table 3; Supplementary Material 6), which explained 61.0% of the variance, consisted of an 8-item *Affective impact*, a 5-item *Perceived risk CVD*, a 4-item *Perceived risk non-communicable diseases (NCD),* a 5-item *Changed self-concept*, a 3-item *CVD group identity*, and a 3-item *Anticipated regret*-factor. The correlations between these factors were small to medium (0.04–0.42) (Table 4).

**Table 3:** Rotated Factor Matrix of the CardiacTM-scale.

	Factor					
Item	**1.** **Affective impact**	**2. Perceived risk CVD**	**3. Changed self-concept**	**4.** **CVD group identity**	**5.** **Perceived risk NCD**	**6. Anticipated regret**
1. When I begin to worry about my heart, I cannot stop worrying.	**0.74**	0.02	0.13	−0.12	0.04	0.15
2. I am worried about having health problems in the future.	**0.59**	0.24	0.16	0.00	0.11	0.12
3. When I begin to worry about my health, I cannot stop worrying.	**0.70**	0.02	0.05	−0.07	0.04	0.17
4. The concerns I have about my cardiac event influence my emotions.	**0.78**	0.23	0.17	0.19	0.05	0.08
5. The concerns I have about my cardiac event influence my daily life.	**0.71**	0.19	0.09	0.24	0.09	0.01
6. Due to my cardiac event, I become more easily emotional.	**0.82**	0.05	−0.01	0.20	0.05	0.04
7. Due to my cardiac event, I am more often anxious	**0.86**	0.09	0.03	0.14	0.04	0.09
8. Due to my cardiac event, I feel more often down	**0.81**	0.09	−0.07	0.09	0.05	0.03
9. It is likely that I will experience a/another heart attack or stroke at some point in my life.	0.12	**0.84**	−0.03	0.06	0.09	0.10
10. I think my chances of having a/another heart attack or stroke in the next ten years are high.	0.11	**0.86**	−0.05	0.04	0.11	0.08
11. With my lifestyle as is, I think my chances of having another heart attack or stroke are small.	−0.02	**−0.49**	0.15	−0.01	−0.14	0.03
12. I think my chances of having another heart attack or stroke are higher than those of other people my age and weight.	0.17	**0.66**	0.14	0.13	0.23	0.01
13. Due to my cardiac event, I rate my risk of a/another heart attack or stroke as higher.	0.24	**0.68**	0.06	0.04	0.18	0.07
14. My role as partner/significant other has become more important to me, due to my cardiac event.	0.12	−0.12	**0.65**	−0.09	0.07	0.06
15. My role as parent has become more important to me, due to my cardiac event.	0.20	0.03	**0.70**	0.07	0.03	0.12
16. Due to my cardiac event, I realize more how important I am to my loved ones.	0.02	0.07	**0.79**	0.05	−0.03	0.09
17. Due to my cardiac event, I realize how precious life is.	0.01	−0.02	**0.65**	0.24	−0.06	0.19
18. Due to my cardiac event, I value myself more.	0.00	−0.04	**0.51**	0.27	−0.04	0.01
19. I don’t feel connected to other heart patients.	−0.10	−0.04	−0.15	**−0.77**	0.03	−0.08
20. Due to my cardiac event, I feel more connected to other heart patients.	0.13	0.08	0.13	**0.92**	0.01	0.10
21. I feel a kinship with other heart patients.	0.18	0.12	0.10	**0.80**	0.00	0.14
22. It is likely that I will experience lifestyle-related diseases at some point in my life.	0.03	0.12	0.04	−0.04	**0.92**	0.05
23. I think my chances that I will experience lifestyle-related diseases in the next ten years are high.	0.04	0.16	0.02	−0.04	**0.92**	0.04
24. Should I continue with my lifestyle as is, I expect to experience health problems.	0.11	0.16	−0.10	−0.02	**0.51**	0.11
25. I think my chances of having lifestyle-related diseases are higher than those of other people my age and gender.	0.11	0.28	0.04	0.08	**0.51**	−0.05
26. Due to my cardiac event, I feel worse about myself if I don’t exercise.	0.19	0.02	0.03	0.03	0.07	**0.80**
27. Due to my cardiac event, I feel worse about myself if I don’t take time to relax.	0.19	0.07	0.20	0.14	0.03	**0.75**
28. Due to my cardiac event, I feel worse about myself if I don’t eat healthily.	0.10	0.10	0.26	0.17	0.06	**0.70**

*Note.* Extraction Method: Principal Axis Factoring. Rotation Method: Varimax with Kaiser Normalization.

CVD = cardiovascular diseases; NCD = non-communicable diseases.

**Table 4:** Inter-factor correlations of the CardiacTM-scale.

	Affective impact	Perceived risk CVD	Changed self-concept	CVD group identity	Perceived risk NCD	Anticipated regret
Affective impact	1					
Perceived risk CVD	0.38**	1				
Changed self-concept	0.25**	0.04	1			
CVD group identity	0.21**	0.16**	0.38**	1		
Perceived risk NCD	0.26**	0.42**	−0.02	0.01	1	
Anticipated regret	0.31**	0.08**	0.29**	0.27**	0.14**	1

*Note.* Pearson correlation. *Correlation is significant at the 0.05 level (2-tailed). **Correlation is significant at the 0.01 level (2-tailed).

CVD = cardiovascular diseases; NCD = non-communicable diseases.

##### 3.3.2.2 Confirmatory factor analysis

**Fig. 1:**
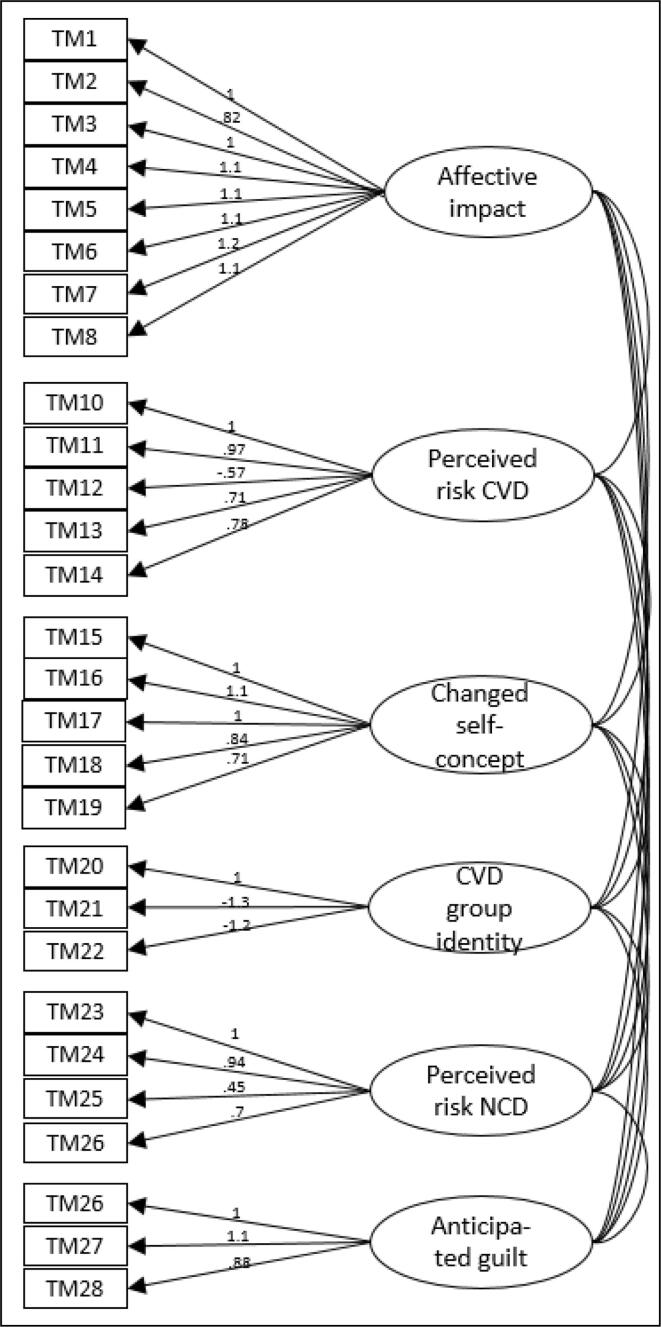
Standardized factor loadings of the six-factor structure with maximum likelihood estimation.

CFA on the six-factor structure is presented in Fig. 1. All standardized factor loadings ranged between 0.45 and 1.3 which is deemed acceptable. The χ^2^-value of 850.80(df = 335) was statistically significant (p < 0.001). The goodness-of-fit statistics showed a CFI = 0.87 and a TLI = 0.85. Lastly, the SRMR value = 0.07 indicated adequate model fit.

To explore whether the six-factor structure had a better fit than the original intended three-factor structure, following the proposed framework of McBride et al. (McBride et al., 2003), we additionally performed CFA on the three-factor structure. Results showed a significant lower model fit compared to the six-factor structure (df = 347; χ^2^ = 1699.00, p < 0.001; CFI = 0.66; TLI = 0.62; SRMR = 0.11.

##### 3.3.2.3 Convergent validity

The results demonstrated moderate to high Pearson correlations (Table 5) of our factors with validated measures of related constructs, indicating that the newly developed scale showed good convergent validity. For example, the factor *Affective impact* demonstrated high convergent validity with the Negative Affect scale of the PANAS (*r* = 0.59; p < 0.01) and the PSWQ (*r* = 0.74; p < 0,01), and the factor *Changed self-concept* showed high convergent validity with the Acceptance scale of the IIQ (*r* = 0.67; p < 0.01). Only the factor *Anticipated regret* demonstrated lower convergent validity (*r* < 0.30) with all comparison measures.

**Table 5:** Convergent validity between factors from CardiacTM-scale and comparison measures.

	**Factors from CardiacTM-scale**					
**Validated measures for convergent validity**	Affective impact	Perceived risk CVD	Changed self-concept	CVD group identity	Perceived risk NCD	Anticipated regret
PANAS NA	0.59**	0.22**	0.20**	0.11*	0.11**	0.29**
PSWQ	0.74**	0.23**	0.13**	0.10**	0.16**	0.26**
ABCD	0.40**	0.69**	0.07	0.07	0.39**	0.14**
IIQ	0.13**	−0.04	0.67**	0.35**	−0.05	0.22**

*Correlation is significant at the 0.05 level (2-tailed). **Correlation is significant at the 0.01 level (2-tailed).

CVD = cardiovascular diseases; NCD = non-communicable diseases.

##### 3.3.2.4 Reliability

The internal consistency (Cronbach’s alpha) was excellent for the factor *Affective impact* (α = 0.93), and good for the factors *Perceived risk CVD* (α = 0.86), *Perceived risk NCD* (α = 0.83), *Changed self-concept* (α = 0.80), *CVD group identity* (α = 0.87), and *Anticipated regret* (α = 0.81). The complete CardiacTM-scale demonstrated good internal consistency (α = 0.88).

#### 3.3.3 Cardiac-induced lifestyle change intention scale

##### 3.3.3.1 Exploratory factor analysis

Of the 15 items of the CardiacLCI-scale, presented in Supplementary Materials 4, two (2 and 11) were initially removed based on non-normality. The resulting 13 items showed good sample adequacy and suitability for EFA (KMO = 0.85; Bartlett’s test of sphericity p < 0.001) when applied to the first half of the dataset. Results of the PAF showed that two factors had eigenvalues over Kaiser’s criterion of 1. Item 10 was subsequently removed based on a communality value of < 0.3, and item 6 was removed because of cross-loadings < 0.2. The final two-factor structure (Table 6; Supplementary Material 6) explained 51.5% of the variance and consisted of a 7-item *Event-related lifestyle change* factor and a 4-item *General healthy lifestyle* factor. The inter-factor correlation was small but significant (*r* = 0.21; p < 0.01).

**Table 6:** Rotated Factor Matrix of the CardiacLCI-scale.

Item	Factor	
	**1.** **Event-related lifestyle change**	**2.** **General healthy lifestyle**
1. I am working hard on improving my lifestyle.	**0.58**	0.01
2. I have made positive changes to my lifestyle.	**0.71**	0.13
3. Due to my cardiac event, I feel the urge to live a healthy lifestyle more.	**0.72**	0.07
4. Due to my cardiac event, I allow myself more time to live a healthy lifestyle.	**0.75**	0.35
5. My cardiac event convinced me that a healthy lifestyle is important for me.	**0.78**	0.09
6. I live a healthier lifestyle now compared to before my cardiac event.	**0.81**	0.11
7. I think of my cardiac event as the start to a new phase in my life*.*	**0.62**	−0.05
8. I am always motivated to live a healthy lifestyle.	0.18	**0.71**
9. As far as I am concerned, my lifestyle is fine as is.	0.02	**0.64**
10. I usually live a healthy lifestyle.	0.23	**0.67**
11. I am easily tempted to do unhealthy things.	0.09	**−0.69**

*Note.* Extraction Method: Principal Axis Factoring. Rotation Method: Varimax with Kaiser Normalization.

##### 3.3.3.2 Confirmatory factor analysis

**Fig. 2:**
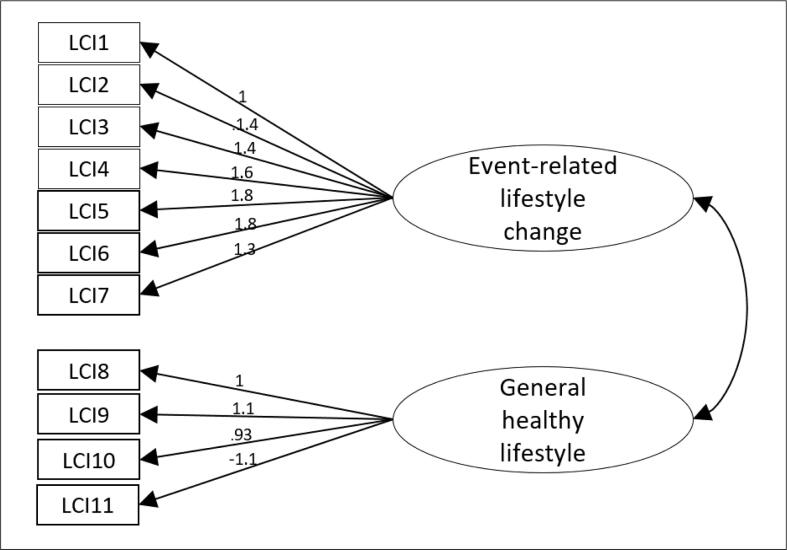
Standardized factor loadings of the two-factor structure with maximum likelihood estimation.

Standardized factor loadings of the two-factor structure with maximum likelihood estimation ranged from 0.93 to 1.8 (Fig. 2), which are deemed sufficient. A χ^2^-value of 1347.27 (df = 55, p < 0.001) suggested poor model fit. However, the goodness-of-fit statistics of the two-factor structure demonstrated an adequate model fit, based on CFI = 0.92 and TLI = 0.90. The SRMR value of 0.08 was slightly below but near to the cut-off score of ≤ 0.08 for adequate model fit. To test the relevance of multiple factors, we performed a CFA on a one-factor structure and found a substantially poorer fit (df = 55; χ^2^ = 1347.27, p < 0.001; CFI = 0.71; TLI = 0.63; SRMR = 0.14).

##### 3.3.3.3 Reliability

The internal consistency (Cronbach’s alpha) was good for *Event-related lifestyle change* (α = 0.86) and sufficient for *General healthy lifestyle* (α = 0.76). The complete CardiacLCI-scale demonstrated good internal consistency (α = 0.81).

##### 3.3.3.4 Association between factors of the two scales

We additionally explored the association between factors of the CardiacTM-scale and factors of the CardiacLCI-scale. All partial correlations between factors of the scales, controlled for age and gender, are presented in Table 7. These results suggest low to high correlations between the factors of the CardiacTM-scale and *Event-related lifestyle change*, of which *Changed self-concept* (*r* = 0.55; p < 0.01) and *Anticipated regret* (*r* = 0.45; p < 0.01) had the highest correlations. The correlations to the *General healthy lifestyle* factor were slightly lower, yet mostly significant. *Perceived risk NCD* has the highest (negative) correlation to this factor (*r* = -0.21; p < 0.01)*.*

**Table 7:** Partial correlation between factors from CardiacTM-scale and CardiacLCI-scale.

	**Factors of CardiacTM**					
**Factors of CardiacLCI**	Affective impact	Perceived risk CVD	Changed self-concept	CVD group identity	Perceived risk NCD	Anticipated regret
Event-related lifestyle change	0.20**	0.09*	0.55**	0.27**	0.02	0.45**
General healthy lifestyle	−0.12**	−0.05	0.14**	0.11**	−0.21**	0.12**

*Note.* Controlled for age and gender. * Correlation is significant at the 0.05 level (2-tailed). ** Correlation is significant at the 0.01 level (2-tailed).

CVD = cardiovascular diseases; NCD = non-communicable diseases.

## 4 Discussion

In the time window following an acute cardiovascular disease (CVD) event, a patient may be more receptive to health behavior advice and more driven to adopt risk-reducing health behaviors (Lawson and Flocke, 2009, Tofler et al., 2015, Coull and Pugh, 2021, Karvinen et al., 2015). The objective of this study was to develop two valid and reliable scales that can be used to conduct empirical research on teachable moments (TMs) in the context of cardiometabolic disorders. The content and construct (factorial) validity of both newly developed scales appeared to be strong. Furthermore, the scales showed good internal consistency reliability and relatively small inter-factor correlations, which confirmed that the factors derived from the factor analyses show meaningful distinctive ability while being intended to measure the same constructs. The types of validity and reliability testing used made it possible to identify and select those items with the best psychometric behaviors.

The first Cardiac Teachable Moment Framework (CardiacTM) scale was developed as a measure to capture whether a cardiac event meets TM characteristics within patients (i.e. affective impact, risk perception, changed self-concept(4)). The final scale consisted of six distinct and reliable (internally consistent) factors, and demonstrated sufficient construct validity and good convergent validity. In total, the scale explained 61.0% of the variance, indicating a good ability to capture variance in perceiving a cardiac event as a TM. The first factor, termed *Affective impact*, consisted of most items that were initially drafted for the affective impact part of the TM framework described by McBride et al. (McBride et al., 2003). The high association with the Negative Affect scale of the PANAS (Thompson, 2007) and the PSWQ (Berle et al., 2011) provided good evidence that the items sufficiently captured this cognitive antecedent of a TM event.

Items that were initially drafted in relation to risk perception, the second concept of the TM framework (McBride et al., 2003), appeared to subdivide across two distinct factors; *Perceived risk CVD* and *Perceived risk non-communicable diseases (NCD).* Although CVD and other NCDs share many risk factors (Peters et al., 2019), patients may differentiate the respective risks. We found that patients who were more aware of their cardiac risk were also more likely to adopt risk-reducing health behaviors after their cardiac event. This positive effect of perceived CVD risk was also noticed by Everett et al. (Alqahtani et al., 2020), who showed that patients with increased risks perception were more likely to adhere to cardiac rehabilitation. These findings may have important implications for the promotion of accurate risk perception in cardiac care.

Items generated for the third concept of the TM framework (McBride et al., 2003), a change in self-concept, appeared to subdivide across three distinct factors. This finding is in accordance with previous studies which have defined self-concept as a broad and comprehensive construct (McBride et al., 2003, Bergner and Holmes, 2000). The factor *Changed self-concept* comprised items on changes in one’s sense of self and meaningfulness, as well as in the importance of certain social roles in life. Hence, we deem this factor to be conceptually the closest to the intended factor of the TM framework (McBride et al., 2003). According to the Social Cognitive Theory, the interpersonal or relational self can instill motivation to adhere to the wishes of salient others (Bandura, 1998). Someone’s health behaviors can thus be influenced by an assessment of the importance of social roles to a person (Stets, 2020). We also found evidence for this phenomenon in the high association between *Changed self-concept* and *Event-related lifestyle change* in the current study, as well as in our previous work (Brust et al., 2021).

Another identified factor was termed *CVD group identity* and consisted of items related to being part of the group of cardiovascular disease patients. A health event can shift someone’s perceived identity towards being an ill person (Feig et al., 1998), and perceiving an illness as part of one’s own identity is in turn associated with optimal disease management behaviors (Van Bulck et al., 2018). An identity shift towards being a CVD patient may increase personal notions of the importance of adopting healthy behaviors, exactly because these behaviors are part of the accepted disease management guidelines. In order to facilitate a TM effect on behavioral change, cardiac rehabilitation programs could therefore place more emphasis on promoting the adoption of a CVD patient identity among their patients.

The final factor, termed *Anticipated regret*, comprised items that ask about perceived feelings of self-regret when partaking in a risk behavior (i.e. lack of exercise, unhealthy eating or not allowing time for relaxation). Evidence supporting the effect of anticipated regret on health behaviors comes from a large *meta*-analysis, in which a strong association was found between anticipated regret after not participating in a protective health behavior and engaging in that behavior (Brewer et al., 2016). Surprisingly, we were obliged to remove the risk behaviors smoking and alcohol consumption based on the factor analysis, which may be partly due to the relatively low number of smokers (5%) and consumers of alcohol (65%) in our sample. However, the process of cognitive dissonance reduction (Festinger, 1957) might also play a role here. According to cognitive dissonance theory, an individual may justify harmful behaviors to themselves in order to decrease cognitive dissonance, a state of mental discomfort that results from concurrent but mutually inconsistent ideas or beliefs (Orcullo and Teo, 2016). As cognitive dissonance has been instrumental in explaining addictive behaviors (Fotuhi et al., 2013, Chiou and Wan, 2007), it may well be worthwhile to further investigate its role on smoking and alcohol consumption following a cardiometabolic diagnosis.

The second scale in the current study, the Cardiac-induced Lifestyle Change Intention (CardiacLCI)-scale, also appeared to be a reliable measurement tool with sufficient content and construct validity. The scale captured 51.5% of the variance in event-induced LCI, a figure comparable to similar scales that measure health behavior change (Woringer et al., 2017, Oliveira et al., 2019). Two distinct and reliable factors could be identified. The first factor, termed *Event-related lifestyle change*, comprised items that measure whether lifestyle change, if it occurs, was specifically instigated by the cardiac event. The relatively robust associations between this factor and factors of the CardiacTM-scale, provides supports our conclusion that truly captured the mechanism of TM in both scales. The other factor, termed *General healthy lifestyle*, comprised items that captured the more stable attitudes towards healthy behavior within patients, those less affected by the event itself. As health behaviors are relatively stable throughout the life course (Burgard et al., 2020), it is possible that patients with high scores on this factor were already living a healthy lifestyle prior to their cardiac event.

Although the current study provides preliminary support for the validity of the two scales, there are two important points that require consideration. First, although stressful health events such as a disease diagnosis are primarily linked to TMs (Xiang, 2016), positive life events such as pregnancy are also associated with sudden lifestyle changes (Phelan, 2010). It is therefore an open question whether affective impact only comprises negative emotions (such as worry and fear) or whether emotions related to positive events (such as being grateful or enthusiastic) also facilitate the TM mechanism. Future studies should consider to also incorporating positive affect when exploring the role of affective impact on behavioral change after life events. Furthermore, according to McBride et al. (McBride et al., 2003) someone’s perceived consequences of engaging in a certain risk behavior, i.e. the expected outcomes, are an important part of the construct risk perception as well as in behavior change theories (Becker, 1974). Although we initially included items that aimed to capture the expected outcomes of health behaviors, these items were deleted based on the factor analysis. During a future optimization of the CardiacTM-scale, this construct thus warrants further attention.

## 5 Future perspectives

The current study has important implications both for research and for cardiometabolic healthcare. As scholars have previously stressed the importance of validated measurement tools within TM research (Lawson and Flocke, 2009, Schnoll et al., 2013), the scales developed in this study may lay the foundation for future research on TMs. Adapted versions of our scales could be employed to explore the potential and underlying mechanisms of life events as TMs, both of which are important to deepening our knowledge concerning life events and behavioral change mechanisms (Lawson and Flocke, 2009). Second, the scales are a first step in the further recognition and utilization of TMs in cardiac and other non-communicable healthcare. Research shows that potential TMs in healthcare more often lead to behavioral change when supported by an appropriate response from healthcare professionals (Flocke et al., 2014, Cohen et al., 2011). Clinicians could therefore employ the scales in a simplified manner during potential TM situations in order to support and guide the conversation with their patients regarding lifestyle change and appropriate follow-up. In summary, in light of the current findings we recommend that the concepts and measurements of behavior change around life events, and related psychological mechanism, should be further developed.

## 6 Strengths and limitations

This study has both strengths and limitations. In our view, the primary strength of this study was the use of a theoretical framework (McBride et al., 2003) during the development of the CardiacTM-scale. A second strength was the use of development stages, as recommended by Boateng et al. (Boateng et al., 2018), including item development, construct development, and construct evaluation stages. Third, the development of scales in collaboration with an expert panel and representatives of the target population resulted in scales that are relatively easy to understand and administer (Olson, 2010). Fourth, rather than solely focusing on one type of validity, our exploration of several types provided us with information on multiple important psychometric properties. Finally, our patient sample consisted of a heterogeneous group with diverse sociodemographic characteristics, suggesting that our results are likely to be generalizable to the broader CVD patient population.

The most important limitation of our study was that we did not fully reach the threshold for good model fit in both scales, although some scholars suggest that statistics slightly below the threshold are sufficient. Another limitation was the use of a cross-sectional design, which precluded the exploration of other tests such as test–retest reliability (Boateng et al., 2018). A third limitation was the relatively small sample size, although the sample was sufficient for scale development (Boateng et al., 2018, Clark and Watson, 1995, Gunawan et al., 2021). A fourth limitation was that the effect of time since most recent cardiac event was not taken into account. As TMs may be time-dependent, future studies should consider exploring the time factor around TMs. The final limitation was the relatively young average age (59 years) of our sample population. As younger individuals may face different challenges compared to older individuals, some scholars recommend treating younger cardiac patients as a specific population (Journiac et al., 2020).

## 7 Conclusion

We developed two scales for the purposes of TM research and provide evidence supporting the reliability and validity of these scales. These easy-to-administer scales can be used by researchers to gain a better understanding of life events as potential TMs, as well as by clinicians to foster a conversation about lifestyle during cardiac rehabilitation or following other life events.

### CRediT authorship contribution statement

**Michelle Brust:** Conceptualization, Methodology, Formal analysis, Investigation, Writing – original draft, Visualization. **Winifred A. Gebhardt:** Conceptualization, Methodology, Formal analysis, Writing – review & editing, Supervision. **Nadine A.E. van der Voorde:** Investigation, Writing – review & editing. **Mattijs E. Numans:** Writing – review & editing, Supervision. **Jessica C. Kiefte-de Jong:** Conceptualization, Methodology, Formal analysis, Writing – review & editing, Supervision, Project administration, Funding acquisition.

## Declaration of Competing Interest

The authors declare that they have no known competing financial interests or personal relationships that could have appeared to influence the work reported in this paper.
