# Supplementary material for: The development and validation of scales to measure the presence of a teachable moment following a cardiovascular disease event
Source: Prev Med Rep. 2022 Jun 27;28:101876. doi: 10.1016/j.pmedr.2022.101876 (PMC9254119; doi:10.1016/j.pmedr.2022.101876)
Supplement: Supplementary data 1 [file mmc1.docx]

# Supplementary Material 1: identification of domains

## Cardiac Teachable Moment Framework scale

Keywords; teachable moment, health behavior change, lifestyle, risk perception, perceived risk, perceived susceptibility, vulnerability, outcome expectancy, affective impact, affect, affective response, emotions, self-concept, social role, self-identity, social identity, group identity, cardiovascular disease, cardiovascular disease patients, heart attack, myocardial infarction, acute coronary event, cardiac event, cardiovascular disease event.

The first teachable moment (TM) characteristic *risk perception* is an important factor in behaviour change theories such as the Health Belief Model, and serves as an explanation for the motivation to comply to risk reducing health behaviours in response to a threat(1). The majority of studies that assess risk perception use the assessment of perceived level of vulnerability or susceptibility for acquiring CVD and thoughts about seriousness or severity when getting CVD. We followed these assessments and included items that capture perceived level of susceptibility for acquiring cardiovascular diseases (CVD), as well as thoughts about that being serious or severe(2-5). In addition, we included items that capture someone’s perceived risk relative to others of similar age and sex(6, 7). Because perceived risk of acquiring other non-communicable diseases (NCDs) is also often assessed in studies on risk perception(8-10), we added perceived risk for other NCDs as an additional subdomain in our scale as well. Lastly, as McBride et al(11) specifically stress the importance of an ‘increase’ in risk perception as a mediating factor, we additionally included items that assessed the extent to which the cardiac event has *altered* perceived risk.

The second TM characteristic *affective impact* plays an important role for a life event being perceived as significant and meaningful enough to prompt behavior change(11). Emotionally loaden contexts or events could have a cueing effect on behavioral decisions(12), such as the decision to adopt protective health behaviors. Life or health events that have a higher impact on emotions and affect are more frequently perceived as a TM(11, 13). First, level of health-related worry is frequently assessed as a subdomain of affective impact(13). In concordance to McBride et al

(9), we assessed not only level of worry for acquiring further cardiac health problems, but also level of worry for acquiring other NCDs in the future. Second, negative emotions such as fear and sadness raise concern about health problems and increase motivation to eliminate health risks by adopting risk-reducing behaviors(13). The extent to which negative emotions are experienced are therefore often assessed when scholars investigate the affective impact of an event. Hence, we included negative affect in our items as well.

*Changed self-concept* is the third characteristic of a TM event(11). Self-concept is an umbrella term for one’s perception about one’s own position in the world and that relative to others(11, 14). For example, one’s self-concept is frequently based on social roles (e.g. role as a partner) and on how important these are for a person(15). Behavior is often in accordance with these roles and depends on which role is most salient at the time(15), as well as on social expectations such as role obligation and stigmatization. For example, becoming a patient could be accompanied by role expectations from others that those who are ill should avoid risky health behaviors(9). We therefore included items that comprise various different social roles (such as role as a partner), and ask for perceived stigmatization regarding one’s behaviors. In addition, researchers who measure self-concept in the context of healthcare often regard it as illness identity, i.e., the extent to which someone identifies themselves as a patient with an illness(16). We therefore added identity as a CVD patient as a subdomain as well. Another study that explored a lung cancer diagnosis as a TM, captured self-concept by asking participants to rate the extent to which smoking fits with their identity(9). A change in self-concept often goes along with re-evaluating the extent to which certain lifestyle behaviors fits with the new perceived self(17). Similarly, we included lifestyle-related identity as an additional subdomain. Lastly, we included items that capture a *shift* in future/possible self, feelings of self-worth, and body image, since these concepts are also frequently linked to the concept of self-concept or identity(18-20).

### Cardiac-induced Lifestyle Change Intention scale

Keywords; teachable moment, health behavior change, lifestyle, lifestyle change intention, adapting lifestyle, healthy lifestyle, cardiovascular disease, cardiovascular disease patients, heart attack, myocardial infarction, acute coronary event, cardiac event, cardiovascular disease event.

The vast majority of researchers who assessed the occurrence of TM only measured intention for behaviour change as the main outcome variable. For example, McBride et al(9) merely applied a one-item measure which assessed participants’ intentions to quit smoking in the next six months. We therefore included items that capture intention to change lifestyle and intention to follow a healthy lifestyle in our scale as well. However, a TM is generally conceptualized as increased behaviour change intentions instigated by an event itself(21). Most studies that investigated TMs took motivation for behaviour change into account, but did not focus on the actual health event as the underlying cause. An exception is the study by Mazanec et al(22) in which items were included that assessed the extent to which the event itself raised awareness of a participants’ health risk and the extent to which the event made them change what they do to take care of their own health. We therefore incorporated items that specifically ask for the impact of the cardiac event on lifestyle and attitude towards lifestyle.

# References

1. Becker MH. The Health Belief Model and Sick Role Behavior. Health Education Monographs. 1974;2(4):409-19.

2. Ammouri AA, Neuberger G. The Perception of Risk of Heart Disease Scale: development and psychometric analysis. J Nurs Meas. 2008;16(2):83-97.

3. Everett B, Salamonson Y, Rolley JX, Davidson PM. Underestimation of risk perception in patients at risk of heart disease. Eur J Cardiovasc Nurs. 2016;15(3):e2-9.

4. Shiloh S, Wade CH, Roberts JS, Alford SH, Biesecker BB. Associations between risk perceptions and worry about common diseases: a between- and within-subjects examination. Psychol Health. 2013;28(4):434-49.

5. Woringer M, Nielsen JJ, Zibarras L, Evason J, Kassianos AP, Harris M, et al. Development of a questionnaire to evaluate patients' awareness of cardiovascular disease risk in England's National Health Service Health Check preventive cardiovascular programme. BMJ Open. 2017;7(9):10.

6. Davidson PM, Salamonson Y, Rolley J, Everett B, Fernandez R, Andrew S, et al. Perception of cardiovascular risk following a percutaneous coronary intervention: a cross sectional study. Int J Nurs Stud. 2011;48(8):973-8.

7. Park ER, Ostroff JS, Rakowski W, Gareen IF, Diefenbach MA, Feibelmann S, et al. Risk perceptions among participants undergoing lung cancer screening: baseline results from the National Lung Screening Trial. Ann Behav Med. 2009;37(3):268-79.

8. Hay JL, Ostroff J, Burkhalter J, Li Y, Quiles Z, Moadel A. Changes in cancer-related risk perception and smoking across time in newly-diagnosed cancer patients. J Behav Med. 2007;30(2):131-42.

9. McBride CM, Blocklin M, Lipkus IM, Klein WMP, Brandon TH. Patient's lung cancer diagnosis as a cue for relatives' smoking cessation: evaluating the constructs of the teachable moment. Psycho-Oncol. 2017;26(1):88-95.

10. Rhee JS, Davis-Malesevich M, Logan BR, Neuburg M, Burzynski M, Nattinger AB. Behavior modification and risk perception in patients with nonmelanoma skin cancer. Wmj. 2008;107(2):62-8.

11. McBride CM, Emmons KM, Lipkus IM. Understanding the potential of teachable moments: the case of smoking cessation. Health Educ Res. 2003;18(2):156-70.

12. DeSteno D, Gross JJ, Kubzansky L. Affective science and health: the importance of emotion and emotion regulation. Health Psychol. 2013;32(5):474-86.

13. McBride CM, Puleo E, Pollak KI, Clipp EC, Woolford S, Emmons KM. Understanding the role of cancer worry in creating a “teachable moment” for multiple risk factor reduction. Social Science & Medicine. 2008;66(3):790-800.

14. Bergner RM, Holmes JR. Self-concepts and self-concept change: A status dynamic approach. Psychotherapy: Theory, Research, Practice, Training. 2000;37(1):36-44.

15. Stets J, Trettevik. 3 Emotions in Identity Theory. 2020.

16. Oris L, Rassart J, Prikken S, Verschueren M, Goubert L, Moons P, et al. Illness Identity in Adolescents and Emerging Adults With Type 1 Diabetes: Introducing the Illness Identity Questionnaire. Diabetes Care. 2016;39(5):757-63.

17. Kearney MH, O'Sullivan J. Identity shifts as turning points in health behavior change. West J Nurs Res. 2003;25(2):134-52.

18. Barreto ML, Frazier LD. Coping With Life Events Through Possible Selves. Journal of Applied Social Psychology. 2012;42(7):1785-810.

19. Meijer E, Gebhardt WA, Van Laar C, Kawous R, Beijk SC. Socio-economic status in relation to smoking: The role of (expected and desired) social support and quitter identity. Soc Sci Med. 2016;162:41-9.

20. Fernández-Bustos J, González-Martí I, Jordán O, Papathomas A. Understanding the relationship between physical activity and physical self-perception in adolescent females: The role of body image. International journal of sport psychology. 2016;47:373-88.

21. Lawson PJ, Flocke SA. Teachable moments for health behavior change: A concept analysis. Patient Educ Couns. 2009;76(1):25-30.

22. Mazanec SR, Flocke SA, Daly BJ. Health Behaviors in Family Members of Patients Completing Cancer Treatment. Oncol Nurs Forum. 2015;42(1):54-62.
