# Supplementary material for: The development and validation of scales to measure the presence of a teachable moment following a cardiovascular disease event
Source: Prev Med Rep. 2022 Jun 27;28:101876. doi: 10.1016/j.pmedr.2022.101876 (PMC9254119; doi:10.1016/j.pmedr.2022.101876)
Supplement: Supplementary data 2 [file mmc2.docx]

# Supplementary Material 2: initial item pool

| Cardiac Teachable Moment Framework (CardiacTM)-scale | | | |
| --- | --- | --- | --- |
| **Construct** | **Subdomain** | **Item** | **Source/ adapted from/ inspired by:** |
| **Risk perception** | Perceived susceptibility and severity of CVD | 1. It is likely that I will experience a/another heart attack or stroke at some point in my life. | Woringer et al.(5) & Ammouri et al.(2) |
|  |  | 2. I think my chances of having a/another heart attack or stroke in the next ten years are low. | Woringer et al.(5) |
|  |  | 3. I don’t expect to experience a/another heart attack or stroke. | Woringer et al.(5) & Ammouri et al(2) |
|  |  | 4. I would find it serious to experience another heart attack or stroke. | Shiloh et al.(4) |
|  |  | 5. Prior to my cardiac event, my lifestyle was bad for my heart. | New item |
|  |  | 6. My lifestyle, as is, is not bad for my heart. | Ammouri et al.(2) |
|  |  | 7. With my lifestyle as is, I think my chances of having another heart attack or stroke are small. | McBride et al.(9) |
|  |  | 8. With a healthier lifestyle. I think my chance of having another heart attack or stroke is low. | McBride et al.(9) |
|  |  | 9. A healthy lifestyle has little impact on my chance of another heart attack or stroke. | Woringer et al.(5) |
|  | Perceived relative risk | 10. I think my chances of having another heart attack or stroke are higher than those of other people my age and weight. | Davidson et al(6) |
|  |  | 11. I think my chances of having lifestyle-related diseases are higher than those of other people my age and gender. | Davidson et al(23) |
|  | Perceived susceptibility and severity of NCD | 12. It is likely that I will experience lifestyle-related diseases at some point in my life. | Woringer et al.(5) |
|  |  | 13. I think my chances that I will experience lifestyle-related diseases in the next ten years are low. | Woringer et al.(5) |
|  |  | 14. I don’t expect to experience lifestyle-related diseases. | Woringer et al.(5) & Ammouri et al(2) |
|  |  | 15. I would find it serious to experience lifestyle-related diseases. | Shiloh et al.(4) |
|  |  | 16. Should I continue with my lifestyle as is, I definitely expect to experience health problems. | McBride et al.(9) |
|  |  | 17. With a healthier lifestyle, I think my chance of health problems is low. | McBride et al.(9) |
|  |  | 18. A healthy lifestyle has little impact on my chance of lifestyle-related disorders. | Woringer et al.(5) |
|  | Increase in risk perception after cardiac event | 19. Since my cardiac event, I think my chances of a/another heart attack or stroke are higher. | New item |
|  |  | 20. Since my cardiac event, I think my chances of lifestyle-related diseases are higher. | New item |
|  |  | 21. Since my cardiac event, I see myself as more fragile. | New item |
|  |  | 22. Prior to my cardiac event, I rated my chance of lifestyle-related diseases as low. | New item |
|  |  | 23. Due to my cardiac event, I think my chances of lifestyle-related diseases are higher. | New item |
| **Affective impact** | Level of worry | 24. I am worried about having a heart attack or stroke in the future. | Woringer et al.(5); Berle et al.(23); McBride et al.(9) |
|  |  | 25. When I begin to worry about my cardiac event, I cannot stop. | Berle et al.(23) |
|  |  | 26. I am worried about having health problems in the future. | Woringer et al.(5); Berle et al.(23); McBride et al.(9) |
|  |  | 27. I am worried about the effects of my lifestyle on my health. | New item |
|  |  | 28. When I begin to worry about my health, I cannot stop. | Berle et al.(23) |
|  | Negative affect | 29. Since my cardiac event, I worry more about my health. | New item |
|  |  | 30. The concerns I have about my cardiac event influence my emotions. | Butler et al.(24) |
|  |  | 31. The concerns I have about my cardiac event influence my daily life. | Butler et al.(24) |
|  |  | 32. Since my cardiac event, I become more easily emotional. | New item |
|  |  | 33. Since my cardiac event, I am more often anxious. | Moss-Morris et al.(25) |
| **Changed self-concept** | Change in … |  |  |
|  | Social role | 34. My role as partner/significant other has become more important to me, since my cardiac event. | Okely et al.(26) |
|  |  | 35. My role as parent has become less important to me, since my cardiac event. | Okely et al.(26) |
|  |  | 36. Since my cardiac event, I realize more how important I am to my loved ones. | Okely et al.(26) |
|  |  | 37. My role as employer/employee has become more important to me, since my cardiac event. | Okely et al.(26) |
|  | Perceived stigmatization | 38. Since my cardiac event, I feel others judge me for my lifestyle choices more readily. | New item |
|  |  | 39. Since my cardiac event, I have more feelings of shame due to disapproval by others. | New item |
|  |  | 40. My loved ones are less likely to disapprove my lifestyle after my cardiac event. | New item |
|  |  | 41. Since my cardiac event, I feel others judge me for doing something unhealthy, such as smoking or eating unhealthy, more. | New item |
|  | Illness identity | 42. My illness is part of who I am as a person. | Oris et al.(16) |
|  |  | 43. I see myself as a heart patient. | New item |
|  |  | 44. I refuse to see my illness as a part of who I am. | Oris et al.(16) |
|  |  | 45. I accept that I am someone with an illness. | Oris et al.(16) |
|  |  | 46. I don’t feel connected to other heart patients. | Cameron (27) |
|  |  | 47. Since my cardiac event, I feel more connected to other heart patients. | New item |
|  |  | 48. I feel a kinship with others who experienced a cardiac event. | Cameron (27) |
|  | Identity and lifestyle | 49. Since my cardiac event, I feel less connected to smokers. | Cameron (27) |
|  |  | 50. Since my cardiac event, I feel less connected to people who eat unhealthy. | Cameron (27) |
|  |  | 51. Since my cardiac event, I feel less connected to people who don’t exercise much. | Cameron (27) |
|  |  | 52. Since my cardiac event, I feel less connected to people who drink alcohol. | Cameron (27) |
|  |  | 53. Since my cardiac event, I feel less connected to people who take little time for relaxation. | Cameron (27) |
|  |  | 54. Since my cardiac event, not smoking fits more with who I want to be as a person. | Shadel et al.(28) |
|  |  | 55. Since my cardiac event, eating healthy fits more with who I want to be as a person. | Shadel et al.(28) |
|  |  | 56. Since my cardiac event, exercising fits more with who I want to be as a person. | Shadel et al.(28) |
|  |  | 57. Since my cardiac event, not drinking alcohol fits more with who I want to be as a person. | Shadel et al.(28) |
|  |  | 58. Since my cardiac event, taking more time for relaxation fits more with who I want to be as a person. | Shadel et al.(28) |
|  |  | 59. Since my cardiac event, I feel worse about myself if I smoke. | McBride et al.(9) |
|  |  | 60. Since my cardiac event, I feel worse about myself if I eat unhealthy. | McBride et al.(9) |
|  |  | 61. Since my cardiac event, I feel worse about myself if I exercise little. | McBride et al.(9) |
|  |  | 62. Since my cardiac event, I feel worse about myself if I drink alcohol. | McBride et al.(9) |
|  |  | 63. Since my cardiac event, I feel worse about myself if I take little time for relaxation. | McBride et al.(9) |
|  | Future/possible self | 64. My future self now has a healthier lifestyle than how I saw my future self before my cardiac event.** | New item |
|  |  | 65. The way in which I see myself in the future has changed since my cardiac event. | New item |
|  |  | 66. Since my cardiac event, I see myself as sicker or weaker in the future. | New item |
|  |  | 67. Since my cardiac event, the future image I have of myself has become hazy/dim. | New item |
|  | Feeling of self-worth | 68. Since my cardiac event, I feel like a lesser person.** | New item |
|  |  | 69. Since my cardiac event, I realize more how precious life is. | New item |
|  |  | 70. Since my cardiac event, I value myself more. | New item |
|  | Body image | 71. The way I view my body has not changed since my cardiac event. | New item |
|  |  | 72. I feel less attractive since my cardiac event. | New item |
|  |  | 73. I feel that others consider me more attractive since my cardiac event. | Cheek and Briggs(29) |
|  |  | 74. My appearance has become more important to me since my cardiac event. | Cheek and Briggs(29) |

| Cardiac-induced Lifestyle Change Intention (CardiacLCI)-scale | | |
| --- | --- | --- |
| **Subdomain** | **Item** | **Source/ adapted from/ inspired by:** |
| Lifestyle change intention | 1. I am motivated to improve my lifestyle. | McBride et al.(9) |
|  | 2. I plan to make positive changes in my lifestyle. | Okely et al.(26) |
|  | 3. As far as I am concerned, my lifestyle is fine as is. | New item |
|  | 4. I am working hard on improving my lifestyle. | New item |
|  | 5. For my health, it is not necessary to change my lifestyle. | New item |
| Impact of event on lifestyle | 6. I have made positive changes to my lifestyle. | New item |
|  | 7. Since my cardiac event, I feel the urge to live a healthy lifestyle more. | New item |
|  | 8. Since my cardiac event, I think more often about wanting a healthier lifestyle. | New item |
|  | 9. Since my cardiac event, I think more positively about a healthy lifestyle. | New item |
|  | 10. My cardiac event has not affected my lifestyle. | New item |
|  | 11. If I don’t change my lifestyle, my chances of having another heart attack or stroke increase. | New item |
|  | 12. My cardiac event convinced me that a healthy lifestyle is important for me. | New item |
|  | 13. It feels as my responsibility to live healthier now compared to before my cardiac event. | New item |
|  | 14. Since my cardiac event, I find it more important to live a healthy life. | New item |
|  | 15. Since my cardiac event, I feel more than before that a healthy lifestyle is necessary for my health. | New item |
|  | 16. I think of my cardiac event as the start to a new phase in my life. | New item |

# References

1. Becker MH. The Health Belief Model and Sick Role Behavior. Health Education Monographs. 1974;2(4):409-19.

2. Ammouri AA, Neuberger G. The Perception of Risk of Heart Disease Scale: development and psychometric analysis. J Nurs Meas. 2008;16(2):83-97.

3. Everett B, Salamonson Y, Rolley JX, Davidson PM. Underestimation of risk perception in patients at risk of heart disease. Eur J Cardiovasc Nurs. 2016;15(3):e2-9.

4. Shiloh S, Wade CH, Roberts JS, Alford SH, Biesecker BB. Associations between risk perceptions and worry about common diseases: a between- and within-subjects examination. Psychol Health. 2013;28(4):434-49.

5. Woringer M, Nielsen JJ, Zibarras L, Evason J, Kassianos AP, Harris M, et al. Development of a questionnaire to evaluate patients' awareness of cardiovascular disease risk in England's National Health Service Health Check preventive cardiovascular programme. BMJ Open. 2017;7(9):10.

6. Davidson PM, Salamonson Y, Rolley J, Everett B, Fernandez R, Andrew S, et al. Perception of cardiovascular risk following a percutaneous coronary intervention: a cross sectional study. Int J Nurs Stud. 2011;48(8):973-8.

7. Park ER, Ostroff JS, Rakowski W, Gareen IF, Diefenbach MA, Feibelmann S, et al. Risk perceptions among participants undergoing lung cancer screening: baseline results from the National Lung Screening Trial. Ann Behav Med. 2009;37(3):268-79.

8. Hay JL, Ostroff J, Burkhalter J, Li Y, Quiles Z, Moadel A. Changes in cancer-related risk perception and smoking across time in newly-diagnosed cancer patients. J Behav Med. 2007;30(2):131-42.

9. McBride CM, Blocklin M, Lipkus IM, Klein WMP, Brandon TH. Patient's lung cancer diagnosis as a cue for relatives' smoking cessation: evaluating the constructs of the teachable moment. Psycho-Oncol. 2017;26(1):88-95.

10. Rhee JS, Davis-Malesevich M, Logan BR, Neuburg M, Burzynski M, Nattinger AB. Behavior modification and risk perception in patients with nonmelanoma skin cancer. Wmj. 2008;107(2):62-8.

11. McBride CM, Emmons KM, Lipkus IM. Understanding the potential of teachable moments: the case of smoking cessation. Health Educ Res. 2003;18(2):156-70.

12. DeSteno D, Gross JJ, Kubzansky L. Affective science and health: the importance of emotion and emotion regulation. Health Psychol. 2013;32(5):474-86.

13. McBride CM, Puleo E, Pollak KI, Clipp EC, Woolford S, Emmons KM. Understanding the role of cancer worry in creating a “teachable moment” for multiple risk factor reduction. Social Science & Medicine. 2008;66(3):790-800.

14. Bergner RM, Holmes JR. Self-concepts and self-concept change: A status dynamic approach. Psychotherapy: Theory, Research, Practice, Training. 2000;37(1):36-44.

15. Stets J, Trettevik. 3 Emotions in Identity Theory. 2020.

16. Oris L, Rassart J, Prikken S, Verschueren M, Goubert L, Moons P, et al. Illness Identity in Adolescents and Emerging Adults With Type 1 Diabetes: Introducing the Illness Identity Questionnaire. Diabetes Care. 2016;39(5):757-63.

17. Kearney MH, O'Sullivan J. Identity shifts as turning points in health behavior change. West J Nurs Res. 2003;25(2):134-52.

18. Barreto ML, Frazier LD. Coping With Life Events Through Possible Selves. Journal of Applied Social Psychology. 2012;42(7):1785-810.

19. Meijer E, Gebhardt WA, Van Laar C, Kawous R, Beijk SC. Socio-economic status in relation to smoking: The role of (expected and desired) social support and quitter identity. Soc Sci Med. 2016;162:41-9.

20. Fernández-Bustos J, González-Martí I, Jordán O, Papathomas A. Understanding the relationship between physical activity and physical self-perception in adolescent females: The role of body image. International journal of sport psychology. 2016;47:373-88.

21. Lawson PJ, Flocke SA. Teachable moments for health behavior change: A concept analysis. Patient Educ Couns. 2009;76(1):25-30.

22. Mazanec SR, Flocke SA, Daly BJ. Health Behaviors in Family Members of Patients Completing Cancer Treatment. Oncol Nurs Forum. 2015;42(1):54-62.

23. Berle D, Starcevic V, Moses K, Hannan A, Milicevic D, Sammut P. Preliminary Validation of an Ultra-brief Version of the Penn State Worry Questionnaire. Clin Psychol Psychother. 2011;18(4):339-46.

24. Butler KM, Rayens MK, Wiggins AT, Rademacher KB, Hahn EJ. Association of Smoking in the Home With Lung Cancer Worry, Perceived Risk, and Synergistic Risk. Oncol Nurs Forum. 2017;44(2):E55-e63.

25. Moss-Morris R, Weinman J, Petrie K, Horne R, Cameron L, Buick D. The Revised Illness Perception Questionnaire (IPQ-R). Psychol Health. 2002;17(1):1-16.

26. Okely J, Mason C, Collier A, Dunnachie N, Swanson V. Diagnosis of gestational diabetes: a 'teachable moment'. Diabetic medicine : a journal of the British Diabetic Association. 2019;36(2):184-94.

27. Cameron JE. A Three-Factor Model of Social Identity. Self and Identity. 2004;3(3):239-62.

28. Shadel WG, Mermelstein R. Individual differences in self-concept among smokers attempting to quit: Validation and predictive utility of measures of the smoker self-concept and abstainer self-concept. Ann Behav Med. 1996;18(3):151-6.

29. Cheek JM, Briggs SR. Aspects of Identity Questionnaire (AIQ-IV). Measurement instrument database for the social science. Measurement Instrument Database for the Social Science <http://www> midss org/content/aspects-identity-questionnaireaiq-iv. 2013.
