# Supplementary material for: The development and validation of scales to measure the presence of a teachable moment following a cardiovascular disease event
Source: Prev Med Rep. 2022 Jun 27;28:101876. doi: 10.1016/j.pmedr.2022.101876 (PMC9254119; doi:10.1016/j.pmedr.2022.101876)
Supplement: Supplementary data 3 [file mmc3.docx]

# Supplementary Material 3: I-CVI rating for clarity and relevance

**Table 1.** Cardiac Teachable Moment Framework (CardiacTM)-scale: I-CVI rating for clarity and relevance (n=6)

| **Items** | **I-CVI**  **Clarity** | **I-CVI**  **Relevance** |
| --- | --- | --- |
| **Risk perception** |  |  |
| 1. It is likely that I will experience a/another heart attack or stroke at some point in my life. | 1 | 1 |
| 2. I think my chances of having a/another heart attack or stroke in the next ten years are low.*** | 0.83 | 0.83 |
| 3. I don’t expect to experience a/another heart attack or stroke.*** | 1 | 1 |
| 4. I would find it serious to experience another heart attack or stroke.* | 0.66 | 0.66 |
| 5. Prior to my cardiac event, my lifestyle was bad for my heart. | 1 | 1 |
| 6. My lifestyle, as is, is not bad for my heart.** | 0.83 | 1 |
| 7. With my lifestyle as is, I think my chances of having another heart attack or stroke are small. | 0.83 | 1 |
| 8. With a healthier lifestyle, I think my chance of having another heart attack or stroke is low.** | 1 | 0.83 |
| 9. A healthy lifestyle has little impact on my chance of another heart attack or stroke.*** | 1 | 1 |
| 10. I think my chances of having another heart attack or stroke are higher than those of other people my age and weight. | 0.83 | 1 |
| 11. I think my chances of having lifestyle-related diseases are higher than those of other people my age and gender. | 0.83 | 1 |
| 12. It is likely that I will experience lifestyle-related diseases at some point in my life. | 0.83 | 0.83 |
| 13. I think my chances that I will experience lifestyle-related diseases in the next ten years are low.*** | 0.83 | 1 |
| 14. I don’t expect to experience lifestyle-related diseases.* | 0.66 | 0.66 |
| 15. I would find it serious to experience lifestyle-related diseases.* | 0.66 | 0.66 |
| 16. Should I continue with my lifestyle as is, I definitely expect to experience health problems.** | 1 | 1 |
| 17. With a healthier lifestyle, I think my chance of health problems is low.** | 0.83 | 1 |
| 18. A healthy lifestyle has little impact on my chance of lifestyle-related disorders.*** | 1 | 1 |
| 19. Since my cardiac event, I think my chances of a/another heart attack or stroke are higher.** | 1 | 1 |
| 20. Since my cardiac event, I think my chances of lifestyle-related diseases are higher.** | 1 | 1 |
| 21. Since my cardiac event, I see myself as more fragile.** | 0.83 | 1 |
| 22. Prior to my cardiac event, I rated my chance of lifestyle-related diseases as low.*** | 0.83 | 1 |
| 23. Due to my cardiac event, I think my chances of lifestyle-related diseases are higher.*** | 0.83 | 0.83 |
| **Affective impact** |  |  |
| 24. I am worried about having a heart attack or stroke in the future. | 1 | 1 |
| 25. When I begin to worry about my cardiac event, I cannot stop.*** | 0.83 | 1 |
| 26. I am worried about having health problems in the future. | 1 | 1 |
| 27. I am worried about the effects of my lifestyle on my health. | 1 | 1 |
| 28. When I begin to worry about my health, I cannot stop.*** | 0.83 | 1 |
| 29. Since my cardiac event, I worry more about my health. | 1 | 1 |
| 30. The concerns I have about my cardiac event influence my emotions. | 0.50 | 0.83 |
| 31. The concerns I have about my cardiac event influence my daily life. | 0.66 | 1 |
| 32. Since my cardiac event, I become more easily emotional. | 1 | 1 |
| 33. Since my cardiac event, I am more often anxious. | 1 | 1 |
| **Changed self-concept** |  |  |
| 34. My role as partner/significant other has become more important to me, since my cardiac event. | 0.83 | 1 |
| 35. My role as parent has become less important to me, since my cardiac event. | 1 | 1 |
| 36. Since my cardiac event, I realize more how important I am to my loved ones. | 1 | 1 |
| 37. My role as employer/employee has become more important to me, since my cardiac event. | 1 | 1 |
| 38. Since my cardiac event, I feel others judge me for my lifestyle choices more readily. | 1 | 0.83 |
| 39. Since my cardiac event, I have more feelings of shame due to disapproval by others. | 1 | 0.83 |
| 40. My loved ones are less likely to disapprove my lifestyle after my cardiac event.* | 0.83 | 0.66 |
| 41. Since my cardiac event, I feel others judge me for doing something unhealthy, such as smoking or eating unhealthy, more.* | 0.66 | 0.66 |
| 42. My illness is part of who I am as a person.*** | 0.83 | 0.83 |
| 43. I see myself as a heart patient. | 1 | 1 |
| 44. I refuse to see my illness as a part of who I am. | 1 | 0.83 |
| 45. I accept that I am someone with an illness.** | 1 | 1 |
| 46. I don’t feel connected to other heart patients. | 1 | 1 |
| 47. Since my cardiac event, I feel more connected to other heart patients. | 1 | 1 |
| 48. I feel a kinship with others who experienced a cardiac event.** | 1 | 1 |
| 49. Since my cardiac event, I feel more connected to people who don’t smoke. | 1 | 1 |
| 50. Since my cardiac event, I feel more connected to people who eat healthy. | 1 | 1 |
| 51. Since my cardiac event, I feel more connected to people who exercise. | 1 | 1 |
| 52. Since my cardiac event, I feel more connected to people who don’t drink alcohol. | 1 | 1 |
| 53. Since my cardiac event, I feel more connected to people who take time for relaxation. | 1 | 1 |
| 54. Since my cardiac event, not smoking fits more with who I want to be as a person.* | 0.83 | 0.66 |
| 55. Since my cardiac event, eating healthy fits more with who I want to be as a person.* | 0.83 | 0.66 |
| 56. Since my cardiac event, not drinking alcohol fits more with who I want to be as a person.* | 0.83 | 0.66 |
| 57. Since my cardiac event, exercising fits more with who I want to be as a person.* | 0.83 | 0.66 |
| 58. Since my cardiac event, taking more time for relaxation fits more with who I want to be as a person.* | 0.83 | 0.66 |
| 59. Since my cardiac event, I feel worse about myself if I smoke. | 1 | 1 |
| 60. Since my cardiac event, I feel worse about myself if I eat unhealthy. | 1 | 1 |
| 61. Since my cardiac event, I feel worse about myself if I exercise little. | 1 | 1 |
| 62. Since my cardiac event, I feel worse about myself if I drink alcohol. | 1 | 1 |
| 63. Since my cardiac event, I feel worse about myself if I take little time for relaxation. | 1 | 1 |
| 64. My future self now has a healthier lifestyle than how I saw my future self before my cardiac event.** | 0.83 | 0.83 |
| 65. The way in which I see myself in the future has changed since my cardiac event. | 1 | 1 |
| 66. Since my cardiac event, I see myself as sicker or weaker in the future. | 1 | 0.83 |
| 67. Since my cardiac event, the future image I have of myself has become hazy/dim.*** | 0.83 | 0.83 |
| 68. Since my cardiac event, I feel like a lesser person.* | 0.5 | 0.66 |
| 69. Since my cardiac event, I realize more how precious life is.** |  |  |
| 70. Since my cardiac event, I value myself more. | 1 | 1 |
| 71. The way I view my body has not changed since my cardiac event.** |  |  |
| 72. I feel less attractive since my cardiac event. | 1 | 0.83 |
| 73. I feel that others consider me more attractive since my cardiac event.* | 1 | 0.66 |
| 74. My appearance has become more important to me since my cardiac event. | 1 | 1 |

*Note.* Items with * are eliminated based on I-CVI < 0.80, items with ** are adapted based on feedback from expert panel and items with *** are eliminated or adapted based on feedback during the think-aloud sessions.

**Table 2.** Cardiac-induced LCI (CardiacLCI)-scale: I-CVI rating for clarity and relevance (n=6)

| **Items** | **I-CVI**  **Clarity** | **I-CVI**  **Relevance** |
| --- | --- | --- |
| 1. I am motivated to improve my lifestyle.*** | 1 | 1 |
| 2. I plan to make positive changes in my lifestyle.*** | 1 | 1 |
| 3. As far as I am concerned, my lifestyle is fine as is. | 1 | 1 |
| 4. I am working hard on improving my lifestyle. | 1 | 1 |
| 5. For my health, it is not necessary to change my lifestyle.* | 1 | 0.66 |
| 6. I have made positive changes to my lifestyle. | 1 | 1 |
| 7. Since my cardiac event, I feel the urge to live a healthy lifestyle more. | 1 | 1 |
| 8. Since my cardiac event, I think more often about wanting a healthier lifestyle.*** | 0.83 | 1 |
| 9. Since my cardiac event, I think more positively about a healthy lifestyle.*** | 1 | 1 |
| 10. My cardiac event has not affected my lifestyle.** | 1 | 1 |
| 11. If I don’t change my lifestyle, my chances of having another heart attack or stroke increase.* | 0.83 | 0.66 |
| 12. My cardiac event convinced me that a healthy lifestyle is important for me. | 1 | 1 |
| 13. It feels as my responsibility to live healthier now compared to before my cardiac event.** | 1 | 1 |
| 14. Since my cardiac event, I find it more important to live a healthy life.*** | 1 | 1 |
| 15. Since my cardiac event, I feel more than before that a healthy lifestyle is necessary for my health.* | 1 | 0.66 |
| 16. I think of my cardiac event as the start to a new phase in my life. | 1 | 1 |

*Note.* Items with * are eliminated based on I-CVI < 0.80, items with ** are adapted based on feedback from expert panel and items with *** are eliminated or adapted based on feedback during the think-aloud sessions.
