# Supplementary material for: The development and validation of scales to measure the presence of a teachable moment following a cardiovascular disease event
Source: Prev Med Rep. 2022 Jun 27;28:101876. doi: 10.1016/j.pmedr.2022.101876 (PMC9254119; doi:10.1016/j.pmedr.2022.101876)
Supplement: Supplementary data 4 [file mmc4.docx]

# Supplementary Material 4: items resulting from step 1: item development

## Cardiac Teachable Moment Framework (CardiacTM)-scale

*Risk perception*

1. It is likely that I will experience a/another heart attack or stroke at some point in my life.
2. I think my chances of having a/another heart attack or stroke in the next ten years are high.
3. A healthy lifestyle can reduce my chances of having a heart attack or stroke.*
4. Prior to my cardiac event, my lifestyle was bad for my heart.*
5. My lifestyle, as is, is bad for my heart.*
6. With my lifestyle as is, I think my chances of having another heart attack or stroke are small.
7. A healthier lifestyle will make my chances of having another heart attack or stroke smaller.*
8. I think my chances of having another heart attack or stroke are higher than those of other people my age and weight.
9. It is likely that I will experience lifestyle-related diseases at some point in my life.
10. I think my chances that I will experience lifestyle-related diseases in the next ten years are high.
11. A healthier lifestyle will make my chances of having a lifestyle-related disease smaller.*
12. Should I continue with my lifestyle as is, I expect to experience health problems.
13. A healthier lifestyle will make my chances of having health problems smaller.*
14. I think my chances of having lifestyle-related diseases are higher than those of other people my age and gender.
15. Due to my cardiac event, I rate my risk of a/another heart attack or stroke as higher.*
16. Due to my cardiac event, I rate my risk of lifestyle-related diseases as higher.*
17. Due to my cardiac event, I see myself as a more fragile person.*

*Affective impact:*

1. I am worried about having a heart attack or stroke in the future.*
2. When I begin to worry about my heart, I cannot stop worrying.
3. I am worried about having health problems in the future.
4. I am worried about the effects of my lifestyle on my health.*
5. When I begin to worry about my health, I cannot stop worrying.
6. Due to my cardiac event, I worry more about my health.*
7. The concerns I have about my cardiac event influence my emotions.
8. The concerns I have about my cardiac event influence my daily life.
9. Due to my cardiac event, I become more easily emotional.
10. Due to my cardiac event, I am more often anxious.
11. Due to my cardiac event, I feel more often down.

*Changed self-concept:*

1. My role as partner/significant other has become more important to me, due to my cardiac event.
2. My role as parent has become more important to me, due to my cardiac event.
3. Due to my cardiac event, I realize more how important I am to my loved ones.
4. My role as employer/employee has become more important to me, due to my cardiac event.*
5. Due to my cardiac event, I feel others judge me for my lifestyle choices more readily.*
6. Due to my cardiac event, I have more feelings of shame due to disapproval by others.*
7. Due to my cardiac event, I realize how precious life is.
8. Due to my cardiac event, I value myself more.
9. I look at my body in the same way now as always.*
10. Due to my cardiac event, I feel less attractive.*
11. I feel that others consider me more attractive since my cardiac event.*
12. My appearance has become more important to me due to my cardiac event.*
13. The way in which I see my lifestyle in the future has changed due to my cardiac event.*
14. The way in which I see myself in the future has changed due to my cardiac event.*
15. Due to my cardiac event, I see myself as sicker and weaker in the future.*
16. I see myself as a heart patient.*
17. I refuse to see my heart problem as a part of who I am.*
18. I accept that I am someone with a heart problem.*
19. I don’t feel connected to other heart patients.
20. Due to my cardiac event, I feel more connected to other heart patients.
21. I feel a kinship with other heart patients.
22. Due to my cardiac event, I feel more connected to people who exercise.*
23. Due to my cardiac event, I feel more connected to people who take time for relaxation.*
24. Due to my cardiac event, I feel more connected to people who eat healthy.*
25. Due to my cardiac event, I feel more connected to people who don’t smoke.*
26. Due to my cardiac event, I feel more connected to people who don’t drink alcohol.*
27. Due to my cardiac event, I feel worse about myself if I don’t exercise.
28. Due to my cardiac event, I feel worse about myself if I don’t take time to relax.
29. Due to my cardiac event, I feel worse about myself if I don’t eat healthily.
30. Due to my cardiac event, I feel worse about myself if I smoke.*
31. Due to my cardiac event, I feel worse about myself if I drink alcohol.*

## Cardiac-induced Lifestyle Change Intention (CardiacLCI)-scale

1. I am always motivated to live a healthy lifestyle.
2. I plan to live healthier soon.*
3. As far as I am concerned, my lifestyle is fine as is.
4. I am working hard on improving my lifestyle.
5. I have made positive changes to my lifestyle.
6. I sometimes think about improving my lifestyle.*
7. I usually live a healthy lifestyle.
8. I am easily tempted to do unhealthy things.
9. Due to my cardiac event, I feel the urge to live a healthy lifestyle more.
10. Due to my cardiac event, I think more negatively about a healthy lifestyle.*
11. I live a less healthy lifestyle now compared to before my cardiac event.*
12. Due to my cardiac event, I allow myself more time to live a healthy lifestyle.
13. My cardiac event convinced me that a healthy lifestyle is important for me.
14. I live a healthier lifestyle now compared to before my cardiac event.
15. I think of my cardiac event as the start to a new phase in my life.

Note. * excluded in the analyses.
