# Supplementary material for: The development and validation of scales to measure the presence of a teachable moment following a cardiovascular disease event
Source: Prev Med Rep. 2022 Jun 27;28:101876. doi: 10.1016/j.pmedr.2022.101876 (PMC9254119; doi:10.1016/j.pmedr.2022.101876)

# Supplementary Material 5: scree plots

**Figure 1.**Scree plot to identify factor extraction – CardiacTM-scale


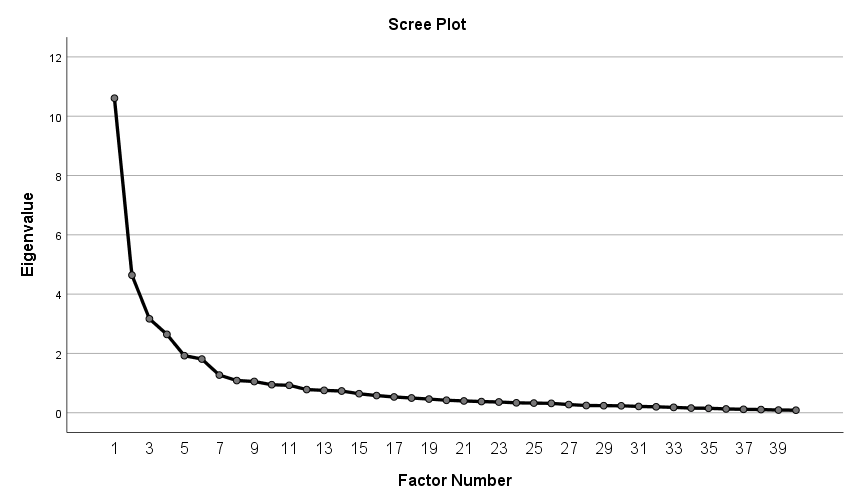


**Figure 2.**
Scree plot to identify factor extraction – CardiacLCI-scale


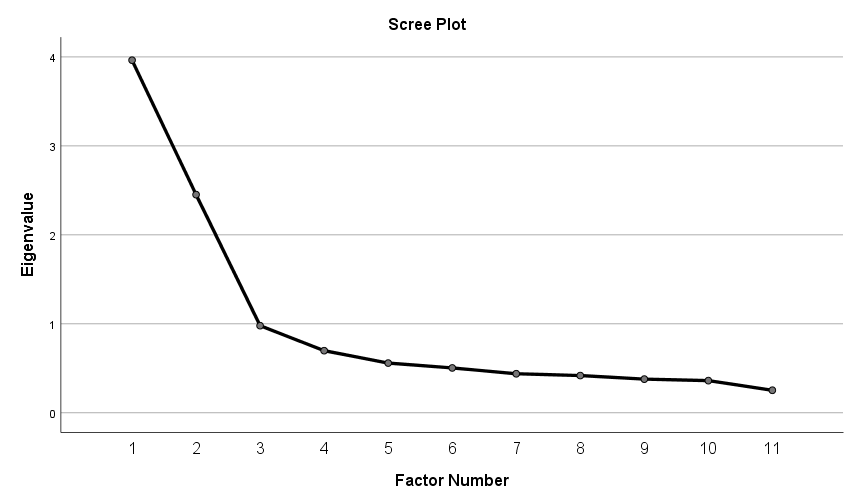

Supplement: Supplementary data 5 [file mmc5.docx]
