# Supplementary material for: The development and validation of scales to measure the presence of a teachable moment following a cardiovascular disease event
Source: Prev Med Rep. 2022 Jun 27;28:101876. doi: 10.1016/j.pmedr.2022.101876 (PMC9254119; doi:10.1016/j.pmedr.2022.101876)
Supplement: Supplementary data 6 [file mmc6.docx]

# Supplementary Material 6: final scales

## Cardiac Teachable Moment Framework (CardiacTM)-scale

Affective impact

- 1. When I begin to worry about my heart, I cannot stop worrying.
  2. I am worried about having health problems in the future.
  3. When I begin to worry about my health, I cannot stop worrying.
  4. The concerns I have about my cardiac event influence my emotions.
  5. The concerns I have about my cardiac event influence my daily life.
  6. Due to my cardiac event, I become more easily emotional.
  7. Due to my cardiac event, I am more often anxious
  8. Due to my cardiac event, I feel more often down.

Perceived risk cardiovascular diseases:

- 1. It is likely that I will experience a/another heart attack or stroke at some point in my life.
  2. I think my chances of having a/another heart attack or stroke in the next ten years are high.
  3. With my lifestyle as is, I think my chances of having another heart attack or stroke are small. (reversed)
  4. I think my chances of having another heart attack or stroke are higher than those of other people my age and weight.
  5. Due to my cardiac event, I rate my risk of a/another heart attack or stroke as higher.

Changed self-concept:

- 1. My role as partner/significant other has become more important to me, due to my cardiac event.
  2. My role as parent has become more important to me, due to my cardiac event.
  3. Due to my cardiac event, I realize more how important I am to my loved ones.
  4. Due to my cardiac event, I realize how precious life is.
  5. Due to my cardiac event, I value myself more.

Cardiovascular disease group identity:

- 1. I don’t feel connected to other heart patients. (reversed)
  2. Due to my cardiac event, I feel more connected to other heart patients.
  3. I feel a kinship with other heart patients.

Perceived risk for non-communicable diseases:

- 1. It is likely that I will experience lifestyle-related diseases at some point in my life.
  2. I think my chances that I will experience lifestyle-related diseases in the next ten years are high.
  3. Should I continue with my lifestyle as is, I expect to experience health problems.
  4. I think my chances of having lifestyle-related diseases are higher than those of other people my age and gender.

Anticipated regret:

- 1. Due to my cardiac event, I feel worse about myself if I don’t exercise.
  2. Due to my cardiac event, I feel worse about myself if I don’t take time to relax.
  3. Due to my cardiac event, I feel worse about myself if I don’t eat healthily.

## Cardiac Lifestyle Change Intention (CardiacLCI)-scale

Event-related lifestyle change

1. I am working hard on improving my lifestyle.
2. I have made positive changes to my lifestyle.
3. Due to my cardiac event, I feel the urge to live a healthy lifestyle more.
4. Due to my cardiac event, I allow myself more time to live a healthy lifestyle.
5. My cardiac event convinced me that a healthy lifestyle is important for me.
6. I live a healthier lifestyle now than before my cardiac event.
7. I think of my cardiac event as the start to a new phase in my life*.*

General healthy lifestyle

1. I am always motivated to live a healthy lifestyle.
2. As far as I am concerned, my lifestyle is fine as is.
3. I usually live a healthy lifestyle.
4. I am easily tempted to do unhealthy things. (reversed)
